# Utilization of routinely collected administrative data in monitoring the incidence of aging dependent hip fracture

**DOI:** 10.1186/1742-5573-4-2

**Published:** 2007-06-07

**Authors:** Reijo Sund

**Affiliations:** 1 National Research and Development Centre for Welfare and Health (STAKES), Health Services and Policy Research, P.O. Box 220, FIN-00531 Helsinki, Finland

## Abstract

Societies are facing challenges as the public health burden increases in tandem with population aging. Local information systems are needed that would allow a continuous monitoring of the incidence and effectiveness of treatments. This study investigates the possibilities of routinely collected administrative data as a data source for hip fracture incidence monitoring in Finland.

The study demonstrates that a straightforward use of register data results in biased estimates for the numbers of hip fractures. An interpretation of hip fractures from the population aging point of view offers an alternative perspective for hip fracture incidence calculation. This enables development of a generalizable method for probabilistic detection of starting points of hip fracture care episodes. Several risk factor and risk population extraction techniques required in register-based data analyses are also demonstrated. Finally, it is shown that empirical evidence suggests that hip fracture incidence is proportional to population level disability prevalence.

In conclusion, Finnish administrative data makes it possible to derive data for rather detailed population level risk factor stratification. Certain limitations of register-based data can be partly avoided by synthesizing data-sensitive methodological solutions during the analysis process.

## Background

Societies are facing challenges as the public health burden increases in tandem with population aging. In order to fulfill the growing need for information for prevention and performance assessment purposes, local information systems are needed that would allow a continuous monitoring of the incidence and effectiveness of treatments for important health problems. However, additional data production requires funding and resources, and it would be beneficial if the required information could be produced using existing administrative data such as hospital discharge registers.

In fact, there is a great deal literature on doing descriptive epidemiology with administrative data [1,2]. The main problem with secondary administrative data is that the straightforward application of standard epidemiological practices may not be feasible, because the data collection cannot be tailored to meet the needs of the exact research problem, as with separate primary data collection. Therefore, it is obvious that the validity of secondary data depends on the research question in mind [3]. In other words, an extra interpretation and preprocessing phase that aims to find an adequate compromise between problem-driven and data-driven approaches becomes an important part of the research process [4]. Even though this phase is commonly encountered while using secondary data in epidemiological studies, it is seldom reported explicitly. In fact, a more coherent report of the results can be achieved by stating that the required compromises were known a priori even if those choices were actually "findings" of the preprocessing phase. The drawback in underreporting is that the data utilization may seem to be easier than it really is. In the worst case, this may lead to uncritical and mechanical repetition of "good examples" in circumstances where the assumptions of those examples are not valid.

This study examines the possibilities of routinely collected Finnish administrative data as a data source for an epidemiologic surveillance system. The basic idea is to demonstrate what kind of issues may require some "rethinking" during a concrete empirical research process that utilizes secondary data. Hip fracture incidence monitoring is used as an example application. First, an example is given of how conventional use of data turns out to produce improper estimates. Because data are not fully compatible with the traditional conceptualization, some alternative theoretical ideas originating from health services and aging research are used as additional links between the problem of hip fracture incidence monitoring and secondary data. After that several pragmatic issues confronted during the empirical research process on hip fracture incidence are examined from a methodological point of view. This study aims to offer practically useful methodological perspectives for the utilization of data by emphasizing the importance of continuous data-sensitive problem solving.

## Analysis

### Conventional approaches for determining the annual numbers of hip fractures by using hospital discharge data

Hip fractures represent a worldwide major public health burden whose impact is expanding as the population ages, with hip fracture incidence rates increasing exponentially with age [5,6]. For an individual, hip fracture is a serious and painful condition that requires and invariably results in acute hospital treatment. Hip fracture is also relatively easy to diagnose (compared with many other health problems), and practically all recorded hip fracture diagnoses (regardless whether principal or secondary) in hospital discharge abstracts reflect hip fracture treatment. Therefore, it can be expected that hospital discharge data are a good source for the identification of hip fracture patients.

#### Pitfalls in the use of hospital discharge data

There are also several known potential pitfalls in using hospital discharge data for calculating the numbers of injures [7-10]. One general nuisance is that only hospitalized injuries are observed. In the case of hip fracture that is not a problem, because virtually all patients with hip fracture require hospital treatment, which results in a discharge record with a hip fracture diagnosis. Another potential pitfall is related to multiple hospitalizations of a single patient. In principle, record linkage allows detection of multiple hospitalizations of the same patient, and the real problem is to define which hospitalizations should be considered as readmissions. There are also other (more or less data-source specific) problems — such as the use of diagnosis codes in the selection of cases — which are typically special cases of common challenges for the use of secondary register data in (epidemiological) research, and require careful data preprocessing that incorporates tacit knowledge formalized in terms of appropriate data abstraction rules [2-4].

#### Hospital discharge data in Finland

In Finland, hospital discharge data are available in the Finnish Health Care Register which records data for all inpatient care discharges in institutions with 24-hour personnel and for outpatient surgical operations. Census data are also collected at the final day of each year in order to capture the ongoing care periods. The register is nationwide, which in international terms is exceptional in that it has such extensive legislative coverage of all public and private service providers. Each record in the register includes data on patient and provider ID-numbers, age, sex, area codes, and diagnosis and operation codes, as well as dates of admission, operation and discharge. Patient ID is a unique identification number given to all Finnish citizens and permanent residents. A system of unique identification numbers has been operating since 1968 and is used in all Finnish registers. Importantly, this system allows complete deterministic record linkage within and between Finnish registers [11]. Data quality is also shown to be good [12-18]. In the case of hip fracture, the completeness is very good and the accuracy of easily measurable variables is at least 95% [18].

#### Register based numbers of hip fractures in Finland

Finnish register data have been previously used as a data source for the calculation of the number of hip fractures [19,20]. For the purposes of this study, all discharge abstracts with a primary or secondary hip fracture diagnosis (10^th ^revision of the International Classification of Diseases diagnoses S72.0, S72.1 and S72.2) from 1998–2002 were identified in the Finnish Health Care Register. The mean number of hospital discharge records (including census records) with hip fracture diagnosis was 14430 per year during 1998–2002 in Finland (Table 1).

**Table 1:** Number of hip fractures in Finland 1998–2002

	1998	1999	2000	2001	2002	Mean
Records with hip fracture diagnosis in the Finnish Health Care Register	14089	13818	14192	14978	15071	14430
The number of patients with hip fracture diagnosis	7817	7661	7643	7924	8030	7815
The number of patients with hip fracture discharge	7575	7465	7425	7706	7854	7605
						
Admissions with hip fracture diagnosis Of which:	13219	12965	13381	14142	14145	13570
- recorded hip fracture operation	5934	5972	6100	6221	6195	6084
- recorded hip fracture operation or at least two years gap to earlier record with hip fracture diagnosis	6742	6631	6702	6924	6937	6787
- recorded hip fracture operation or at least two months gap to earlier record with hip fracture diagnosis	7246	7094	7136	7411	7405	7258
						
Admissions with first record with hip fracture diagnosis in ten years Of which:	5990	5853	5932	6083	6141	6000
- persons aged 50 or more	5551	5413	5543	5644	5667	5564

However, the annual numbers of hip fracture discharge records do not tell the actual number of hip fracture events. A typical care chain for a hip fracture patient consists of acute hospital treatment on a surgical ward and follow-up care on a general ward or in a specialist rehabilitation unit. In addition, a patient may be readmitted for the same fracture after initial discharge to home or to a long-term facility. In order to prevent multiple counting of cases, an individual-based record linkage is recommended for the detection of multiple hospitalizations for the same fracture [10]. This is not a problem with Finnish data, and the previous Finnish studies have identified all hospital discharge records with hip fracture diagnosis and then used calendar year boundaries to exclude multiple hospitalizations of each patient. The corresponding annual number of patients with hip fracture discharge was on average 7605 per year between 1998 and 2002 (Table 1).

Unfortunately, the use of calendar year boundaries makes such an exclusion approach artificial and has no epidemiological justification, because it is clear that calendar year boundaries result in the fact that the related hip fracture free clearance periods (time from the beginning of the year to a first fracture hospitalization of the year) vary per patient. This has two serious consequences. First, it is likely that many patients having their fracture during the final months of each year are erroneously counted as separate cases for two years, because it takes at least four months until the maximum restoration in terms of residential status (hospitalizations) is reached at the population level [21]. Second, one patient may have more than one hip fracture per year resulting in some undercounting. These drawbacks are an indication of need for a more appropriate estimate of the annual number of hip fracture events.

It is reasonable to hypothesize that virtually all recorded hospitalizations with a hip fracture diagnosis combined with hip fracture operation represent a new hip fracture, and that the date of an acute admission for surgery can be used as an estimator for the actual occurrence date of the hip fracture. The mean number of such operations was 6084 per year in Finland during 1998–2002 (Table 1). Unfortunately, this simple definition underestimates the true number of hip fractures. Even though a small number of (incorrectly recorded) re-operations may be erroneously included, the definition excludes patients treated conservatively (non-operatively) as well as patients who die before the operation. It is also known that the recording of hip fracture operations in The Finnish Health Care Register has not been totally complete [18,22]. This means that even some hip fracture related admissions without recorded operations should be considered indications of fresh hip fracture. The problem is to define which admissions reflect fresh hip fractures, and an easy solution is to use a constant hip fracture free clearance period to sort out new admissions from readmissions. If the new admission is defined as a hip fracture related record with no other hip fracture admissions for that individual in the previous two years, the mean number of hip fractures is 6787 per year (Table 1). As the use of long clearance periods may exclude some true subsequent fractures, the numbers of hip fractures were calculated also with a two-month hip fracture free clearance period. With this criterion, the mean number of hip fractures was 7258 per year (Table 1).

In principle, further diagnosis-based exclusion rules could be utilized in order to exclude certain nonstandard cases, such as pathological hip fractures, arthrosis related fractures, and cases with multiple fractures or orthopedic aftercare. However, variations in coding practices partially invalidate the use of such data abstraction rules, which assume recording of secondary diagnoses. In practice, even the clinical criterion of hip fracture may have some regional variation which makes it impossible to obtain a unique estimate for the number of hip fractures using register data.

In any case, following the reasoning described above, it is likely that the mean number of hip fractures is somewhere between 6800–7200 per year in Finland between 1998 and 2002. The estimate for the number of hip fractures in 1998 is 6742–7246. Even the upper limit is significantly smaller than in the earlier Finnish study [20], where the annual number of hip fractures in 1998 was reported to be 7698. The reasons for the small difference in relation to the number of hip fracture discharges in 1998 (Table 1) are not known, but are most probably due to difficulties in reconstruction of the exact operationalization of some inclusion and exclusion criteria used in the earlier study. Also some overestimation is likely to occur because of the artificial definition of fresh hip fracture, as was described above. In conclusion, the earlier study reports a number that seems to be about 10% too large (corresponding to about 700 hip fractures).

### Alternative theoretical approaches

As was demonstrated above, it is difficult to derive adequate estimates for the number of hip fractures even with the careful use of register data. The main problem is that the secondary data are not completely compatible with the theoretical objective of calculating the number of fresh hip fracture events. In other words, it would be beneficial if the theoretical objectives could be modified so that they are in agreement with the limits of the available register data. Fortunately, certain methodological ideas originating from health services and aging research are also useful in epidemiological applications.

#### Care episode approach

The definition for an incident case is a common problem in epidemiologic research. At the conceptual level this means that all events related to the same underlying disorder should be recognized. This is the fundamental idea in the care episode approach [23]. The care episode approach is widely applied in health economics and in health services research. The key point is that the care episode approach offers a sound methodological framework for dealing with multiple hospitalizations in terms of hospital discharge data, and it also provides a sound basis for the measurement of incidence, because an appropriate episode measure combining related hospitalizations is less subject to over- or undercounting of cases [24].

Unfortunately, a sound methodological framework does not automatically solve empirical difficulties [25]. One of the biggest practical problems with linked register data is to determine the starting point of the care episode. In principle, the starting point of a care episode for hip fracture should be rather easy to determine, because the actual event of fracture (or hospitalization following that event) is an obvious index point. However, in practice it is challenging to make a distinction between true subsequent hip fractures and hospitalizations due to ongoing treatment episodes or reoperations in terms of data. In standard practice, it is common to use the first health problem-related event available in the data as an index point. Another widely used approach is to use certain clearance periods to determine the appropriate index points. A third technique is to use external complementary data in determining (the number of) incident cases [26]. Other approaches seem to be rare [27].

Intuitively, it seems to be a good idea to determine an individual's first hip fracture occurrence and consider it as a starting point for a (chronic) care episode. In terms of hospital discharge data this corresponds to the detection of an individual's first hospitalization with recorded hip fracture diagnosis. The problem is that the hip fracture itself is not a chronic condition, but a remediable health deficiency. Therefore, an alternative theoretical interpretation for the first hip fracture is needed.

#### Gompertzian interpretation of aging-related hip fractures

One particularly interesting fact is that hip fracture incidence increases exponentially with age [5]. This kind of functional dependency (Gompertz law) has very strong interpretations from the point of view of population aging [28-30]. In this sense, perspectives from aging research may strengthen the traditional epidemiological interpretations (and vice versa) [31,32]. Biologically, aging can be seen as a complex process occurring stochastically in organs and tissues after reaching adulthood (and mainly after reproductive maturity), which results in irreversible damage accumulation and vulnerability to the failures in maintaining the integrity of tissues and organs [33,34]. A particularly fruitful approach is to consider aging-related events of interest as failures in components of a biological system [35]. An exponential increase of incidence with age means that the intensity of failures is constant (as the probability of failure is constant in time, the cumulative probability of failure is exponential). Therefore, it is not surprising that most aging-related (Gompertzian) conditions are closely connected to (cumulative) alterations in certain tissues or organs.

However, hip fracture is not just an aging-related failure in some single tissue or organ, but is typically the result of an accidental event (such as a fall). In other words, cumulative damage in some tissues (such as bone and muscle) increases the probability of (accidental or pathological) fracture, but it is also likely that some aging-related conditions increase the risk of accident. In this sense, hip fracture seems to capture effects of non-fatal failures in several components of the human body. Among the Gomperzian conditions, only death seems to have a similar multidimensional interpretation as it captures fatal failure(s) in any vital components of human body. In other words, an interesting (methodological) analogy can be seen between death and hip fracture.

It is also obvious that serious but non-fatal failures in components of the human body result in some kind of disabilities. Such disabilities — typically measured in terms of reduced functional capacity and coping with physical activities of daily living — are important risk factors for hip fracture [36,37]. In other words, it is reasonable to hypothesize that the prevalence of such risk factors in the underlying population is related to the incidence of (aging dependent) hip fractures. Following this interpretation, it becomes clear that the occurrence of first aging-dependent hip fracture gives an approximate upper limit for the time of development of critical hip fracture risk factors, because the risk factors must have exceeded the critical level before the event of (low-energetic aging dependent) hip fracture. From this it follows that the first aging-related hip fracture can be seen as an indication of a chronic (disability) condition, and any subsequent hip fractures represent just a nuisance for related population level interpretations. In conclusion, there are theoretical justifications for the determination of the first aging-related hip fracture occurrences, which are in concordance with the restrictions of available hospital discharge data. This interpretation is also of particular importance in a theoretical sense, because it suggests that a commonly available time series of hip fracture incidence may also reflect the more general disability trends of the population, which is a testable hypothesis.

### Calculating the number of hip fractures

So far, the methodological problem of the definition of the hip fracture episode has been reduced to a detection of first aging-related hip fractures in the population (starting points of the first hip fracture care episodes). This is not necessarily a straightforward task in practice, because the available data allow only limited backward follow-up time and it remains unknown whether the first hip fracture found in the data really is the first hip fracture of the person. Probabilistic methods can be used to correct the number of observed persons to the number of persons with the first appearance of a chronic condition [27].

However, if there is a need to identify the persons with a first hip fracture in terms of care episode (instead of just calculating the number of persons with a first hip fracture), it is inconvenient that the actual backward follow-up times (definitions of first hip fractures) vary between persons. In addition, the subsequent hip fracture of a person is biasing interpretations only as far as the risk of subsequent hip fracture is significantly higher than the risk of first aging-related hip fracture. In other words, there may be a cut-off point after which the probability of having a new hip fracture is reduced to the level it would be even without preceding hip fracture, i.e. preceding hip fracture is an "uninformative" predictor of the new hip fracture. By examining the observed and expected probabilities of preceding hip fractures it becomes possible to give justification for a suitable clearance period to be used. Importantly, this probabilistic argument for the definition of the starting points of the care episodes also generalizes easily to other disorders.

#### Probabilistic determination of the starting point of care episode

In order to determine the cut-off point for a clearance period, the first admissions (index points) with the (principal or secondary) diagnosis of hip fracture in 1998–2002 were identified for each patient. To ensure sufficient (backward) follow-up time for each patient, all available discharge records of the hip fracture population from 1987–2002 were obtained from the registers using the unique personal identification numbers as linkage keys. For each index point, backward time to the previous hip fracture admission of the same patient was calculated. Time was measured in months. There were data from the years 1987–2002, so the minimum (backward) follow-up time was 11 years.

**Figure 1:**
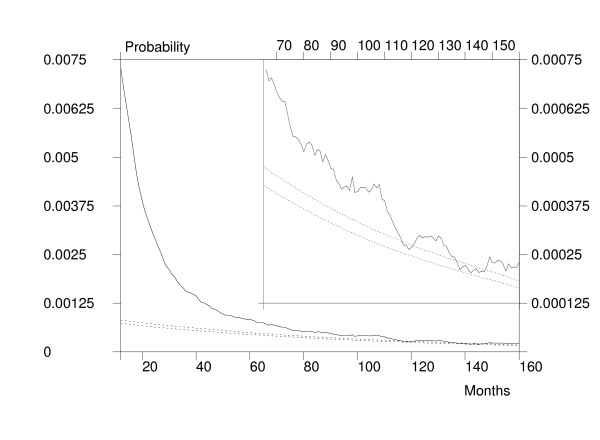
Probability of having a preceding hip fracture as a function of backward time in months from the first fracture in 1998–2002. The smaller picture is a tenfold magnification of the final months. Dotted curves represent the expected probabilities of having a hip fracture (upper curve is calculated with a one-year clearance period and the lower curve with a ten-year clearance period, see text for more information).

The first task was to calculate the expected probabilities of earlier hip fractures in the hip fracture population of interest. The problem here is that reasonable estimates for the incidence of first hip fractures are needed for the calculation. In this study, it was hypothesized that clearance periods between one and ten years may result in a reasonable definition of first hip fracture, and the mean age- and sex-group specific incidences between 1998 and 2002 were calculated by using one year and ten year clearance periods. In order to calculate the actual expected probabilities, the logarithms of the mean hip fracture incidence rates for different age-groups were used in estimating the exponential trend in age-group specific rates by using (log-)linear regression analysis for both sexes separately [38]. This functional relationship allows the interpolation of an incidence rate for persons who are over 40 years old [29]. The individuals with an observed hip fracture were then followed (backwards) in time, with age correspondingly corrected at each time point for all persons in the risk population. With a known age and sex distribution of the risk population, it was possible to predict the expected probability of hip fracture by using the estimated log-linear relationship between age and hip fracture incidence. For ages below 40 years, a constant incidence rate was used in the prediction. These probabilities were then summed across the risk population resulting in an expected number of hip fractures that was finally divided by the size of the risk population, giving an expected (conditional) probability of hip fracture (for each time point). The expected probabilities are shown in Figure 1 using dotted lines. As can be seen, the difference between expected probabilities based on one- and ten-year clearance periods is quite small.

In order to calculate the observed probabilities, a hazard function giving the (conditional) probabilities of having a preceding hip fracture admission as a function of backward time (i.e. time was measured from index admission to a preceding hip fracture admission) in months was estimated nonparametrically using a product-limit estimator, and smoothed using a polynomial moving average. As the estimated hazard function in Figure 1 shows there was an increased and non-stabilized (non-constant) risk for an admission involving hip fracture occurring about 120 months after the previous admission related to hip fracture and the observed risk remains higher than expected in the ten-year period. If a more formal way is needed to determine where the lines meet, it is straightforward to calculate confidence intervals for the hazard function. In conclusion, it seems that a clearance period between seven and ten years is needed until the risk is reduced to the same level as that without a preceding hip fracture. In this study, a conservative ten-year clearance period was selected. The ten-year criterion has also been used in other studies [39].

#### Estimating the number of new hip fractures with limited data

It is unfortunate that no shorter than a ten-year criterion seems to be suitable for hip fractures, because in most countries there may not be the required data available for such a long backward follow-up period. However, if an estimate for the overall number of first-ever hip fractures is enough, and there is no need to identify which patients actually had their first hip fracture, data with limited backward follow-up can also be used.

**Figure 2:**
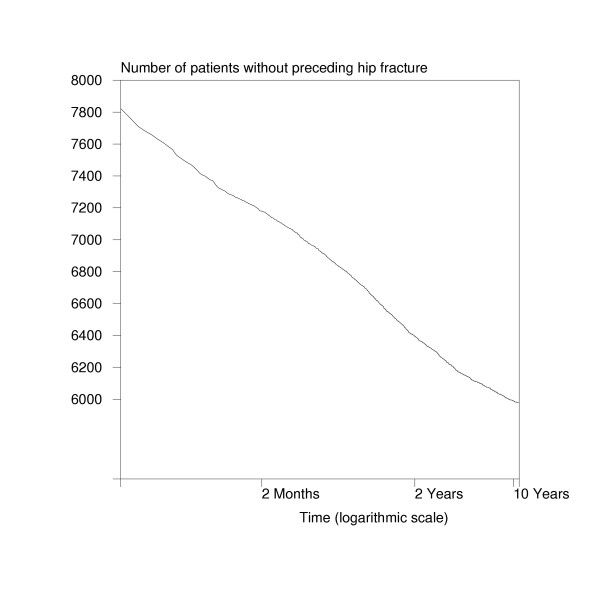
Number of patients without preceding hip fracture as a function of backward time in 1998 in Finland.

The numbers of patients without a preceding hip fracture as a function of backward time in the logarithmic scale for 1998 are drawn in Figure 2. As can be seen, there is almost a linear relationship between logarithmic time and the number of patients without a preceding hip fracture. In other words, even data with quite a short backward follow-up time allow the estimation of such a linear trend resulting in a reasonable prediction for the number of hip fracture patients at the ten year cut-off point (or at any other desirable cut-off point) for a clearance period. For example, if only one year data for backward follow-up are available, the idea is to calculate time from index admission to previous fracture or to the beginning of (backward) follow-up. Then for each day (preceding the index admission) the number of patients with a longer time distance for the previous fracture is calculated. Finally, a logarithmic transformation is applied to the day variable, and a linear regression model (where the number of fractures is a response and constant and logarithmic day are explanatory variables) is estimated. Predictions from this linear model can be easily extrapolated to any cut-off point of interest.

### Risk factor extraction

Using Finnish register data it is feasible to identify hip fracture patients who have their first aging-related hip fracture in the sense described above. This makes it possible to determine the status of certain hip fracture risk factors available in the data — such as age, sex, institutionalization, urbanity, season and year [40-45] — in relation to these patients. Methodologically, three different risk factor extraction techniques can be separated: internal, external, and empirical.

#### Internal extraction

Internal extraction corresponds to the use of data abstraction rules within the register data. For example, using the Finnish data it is not enough to determine the index hip fracture admission, but the actual day of hip fracture must be inferred using appropriate algorithms. It is obvious that the care episode related to hip fracture starts from the first contact with the health care system after the actual event of fracture. However, the index admission could be for a long stay in residential care, while the diagnosis referred to an accident that happened near the discharge day, or the index admission could be for a surgical period, while the actual accident had happened before the admission. After the detection of initial surgical admission, more abstraction rules can be developed. For example, the most accurate diagnoses can be extracted from the data corresponding to the surgical treatments following the fractures, since those are the first ones based on x-ray and physical examinations. For the purposes of this study, a person was classified as an institutionalized long-term care patient if he or she was admitted to the surgical ward from some institution providing inpatient care, and if he or she also had a recorded administrative decision for long-term care or had received inpatient care for more than six months during the year preceding the fracture.

#### External extraction

In external extraction, variables that link individuals to aggregate levels are first internally extracted and then external data that describe aggregate units are linked to each individual. For example, for the purposes of this study, the area code (municipality) at the index admission was used to classify each patient as rural or urban (including semi-urban areas) using the official grouping defined by Statistics Finland (rural municipalities are those municipalities in which less than 60 per cent of the population lives in urban settlements). In general, any other patient characteristic attributable to area-specific phenomena could also be used here instead of urbanity.

#### Empirical extraction

**Figure 3:**
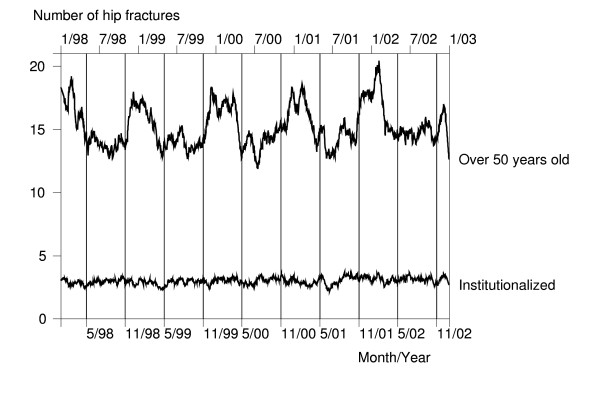
Daily numbers of hip fractures in Finland 1998–2002.

In empirical extraction, preliminary analyses of the available data are used to justify the definitions of variables and data abstraction rules required in the internal extraction. For example, it is not obvious what kind of definition for seasons should be used. This problem can be solved when using the Finnish register data, which allow accurate calculation of daily numbers of hip fractures. After smoothing out the random variation in absolute numbers by a moving-average technique, the mean of daily numbers of the new hip fractures was 15.2 for persons aged 50 or older (Figure 3). There was small but clear seasonal variation so that 53.5 per cent of fractures occurred during the winter/spring season (from November to April) compared to 46.5% during the summer/fall season (from May to October). Data also revealed that there had been some very "hazardous" days during the winter season, but seasonal variation was almost completely due to non-institutionalized persons (Figure 3). Using these results (of preliminary analyses) in the definition of seasons is an example of empirical extraction. In fact, the method developed above for the definition of first aging-related hip fracture is another example of empirical extraction.

### Risk population data

For incidence calculations data on risk population are also needed. Typically, the official population figures are available in administrative databases with stratification according to age, sex and area of living. In this study, population figures (taken on the last day of the years 1997–2002) in 5-year groups were obtained for each municipality (local administrative unit in Finland) from the Social and Health Service Statistical Database (SOTKA). Municipality works as an aggregate unit in external extraction, and also allows easy determination of population figures for any combination of these basic units.

#### Risk population for internally extracted risk factors

It is more difficult to determine risk population for internally extracted risk factors. In this study, long-term institutional care was one risk factor of interest. Fortunately, data from the Finnish Health Care Register can be used to calculate the total numbers of clients in long-term institutional care on the last day of the years 1997–2002, since the register includes all inpatient hospital and nursing home care in Finland. This individual level data can be easily aggregated to appropriate groups (such as stratification by age, sex and municipality), and further subdivision of population figures according to long-term institutional care is possible. However, during the research process it turned out that the calculation technique used in official statistics concerning institutional care was inappropriate for the purposes of epidemiological studies. Therefore, the significantly downwards biased numbers were corrected using a technique reported elsewhere [46].

This procedure finally resulted in simultaneously observed risk populations on the final day of the years 1997–2002 with stratification by sex, age, urbanity, and institutionalization. The derivation of such exceptionally detailed nationwide population figures was possible because of common aggregate units (municipalities) in the databases and registers.

#### Trends for risk populations

Observed risk populations on the final day of the years of interest are not good approximations for the size of the risk population (or the follow-up time for risk population). A common method is to interpolate the mean population by using observed census data on two consecutive years (approximating also the follow-up time of the risk population, if measured in person years). This method can be generalized by modeling the trends extractable from observed risk populations. In this study, regression models with the constant, year and square(year) as regressors were fitted for each group with a stratification by sex, age, institutionalization, and urbanity (with age groups 50 to 64, 65 to 74, 75 to 84 and 85+). These models were then used in the approximation of the required risk populations varying by year and–importantly–by (empirically extracted) seasons, too. Finally, there were the observed numbers of hip fracture events and the follow-up times (in person years) for 320 groups.

#### Limitations of risk population data

It is well known that an accurate number of the population at risk is an essential requirement for incidence calculations [47]. In this study it was possible to derive population-based denominators for each group of interest. These denominators are desirable in terms of accuracy, but not perfect, because an assumption of a stable population (no short-term fluctuations in migration or mortality) is needed for interpolating census day population figures to appropriate mean follow-up times.

In addition, as a ten-year clearance period was used in this study, a person is not at risk of a new hip fracture for the 10 years following a hip fracture and should be — in principle — left out of the risk population. However, this correction was not done in this study, because adequate (group-specific) hip fracture prevalence data were not available. The bias resulting from keeping the prevalent pool in the risk population was considered to be insignificant, as the number of hip fractures is very small in relation to the population in younger age-groups and mortality following the hip fracture is very high for the older age-groups. In fact, relative bias [1-(uncorrected incidence/corrected incidence)] of mean incidence seemed to be less than 2%. Moreover, even if some group-specific bias exists, the direction is towards conservative estimates (underestimation rather than overestimation of incidences).

In conclusion, there is still room for improvement in deriving risk population data. However, the problems are insignificant in terms of the "iceberg phenomenon" involved in the denominator problem [47], and there is no reason to believe that the data used in this study would not reflect the true epidemiology of hip fracture.

### Hip fracture incidence

After data preparation, it is simple to examine the univariate effects of population-level risk factors using standard methodology. It is quite straightforward to extend such analyses for other stratifications of interest, such as area specific estimates (possibly requiring empirical-Bayes estimation) revealing regional variation in hip fracture incidence. Results of such basic analyses are reported elsewhere [48]. Only exemplary results on the simultaneous effects of risk factors with an interpretation offering a new epidemiological perspective for hip fracture are given here.

In standard practice, only a few risk factors are typically considered simultaneously in population level incidence studies. Risk factors of interest are included into the model, and estimates are interpreted as independent effects (effects adjusted for other risk factors in the model). The problem is that there may be complicated interactions between the risk factors, which distort straightforward interpretations.

In this study, the Poisson-regression model was used in the simultaneous examination of several risk factors. Other analyses have indicated that the (long-lasting) increase in age-adjusted incidence had recently stabilized in Finland, with the age-adjusted incidence almost constant between 1999 and 2002 [48]. As the interest in this study was not in secular trends of incidence but in the effects of risk factors, the final Poisson regression model was estimated using the combined data from 1999 to 2002. All main effects (sex, age, institutionalization, urbanity, and season) and statistically significant interactions up to the third degree were included in the model. The goodness of fit was acceptable in terms of Pearson chi-square statistic. Because there were a lot of significant interactions between risk factors, it was rather difficult to report all relevant estimates in easily interpretable form. Therefore, the group-specific rates adjusted for all other factors were calculated by anti-logging the corresponding linear predictors (without the offset) based on the estimated model. These adjusted rates represent the systematic effects extracted from the data by the model. Overlapping confidence intervals of two groups indicate that there is no statistically significant difference between rates in these groups.

#### Alternative epidemiological perspective for hip fracture incidence

The results in Table 2 show that there is a higher hip fracture incidence among older persons and women than among younger persons and men managing at home. However, there is no significant effect of sex on incidence among institutionalized persons. The incidence is higher during the winter time for all persons with a functional status potentially good enough to allow for walking outdoors (non-institutionalized under age 85). Urbanization is not associated with significantly higher hip fracture incidence in Finland. As it is known that institutionalization is a sign of reduced coping with daily activities [49] and that women tend to have more functional limitations and physical disability than men as age increases [50], the results are in concordance with the hypothesis suggesting that hip fracture incidence is proportional to the prevalence of disability related hip fracture risk factors in the underlying population.

**Table 2:** Adjusted incidence rates per 100000 person years for hip fracture in Finland 1999–2002

	Urban				Rural			
		
	Winter		Summer		Winter		Summer	
				
	Rate	95% CI	Rate	95% CI	Rate	95% CI	Rate	95% CI
Non-institutionalized persons								
Men 50–64	62	57, 67	49	45, 53	46	40, 53	36	31, 42
Women 50–64	45	41, 49	35	32, 39	46	39, 53	36	31, 42
Men 65–74	191	178, 205	158	147, 170	176	159, 195	145	131, 161
Women 65–74	223	211, 237	185	174, 197	234	214, 254	193	177, 211
Men 75–84	608	574, 644	503	474, 533	561	516, 609	464	427, 504
Women 75–84	966	932, 1002	799	769, 830	925	878, 974	765	725, 807
Men 85+	1797	1668, 1936	1622	1504, 1748	1858	1680, 2054	1676	1515, 1854
Women 85+	2805	2693, 2922	2531	2426, 2640	2567	2423, 2720	2316	2184, 2457
								
Institutionalized persons								
Men 50–64	1380	1074, 1773	1277	993, 1643	916	689, 1218	848	638, 1128
Women 50–64	1214	908, 1622	1123	840, 1503	1096	795, 1511	1014	735, 1400
Men 65–74	1688	1437, 1983	1646	1400, 1934	1387	1149, 1674	1352	1120, 1633
Women 65–74	2018	1775, 2294	1967	1730, 2237	1884	1616, 2196	1836	1575, 2142
Men 75–84	2106	1883, 2355	2053	1836, 2296	1735	1511, 1992	1691	1473, 1942
Women 75–84	2495	2332, 2670	2432	2272, 2604	2134	1946, 2339	2080	1897, 2282
Men 85+	2330	2066, 2628	2480	2200, 2795	2152	1861, 2489	2290	1981, 2647
Women 85+	2529	2376, 2692	2691	2531, 2861	2068	1894, 2257	2200	2017, 2400

In order to test this hypothesis, a correlation was calculated between the nationwide data on the prevalence of outdoor walking ability — being a strong disability-related risk factor for hip fracture [51] — of persons aged 65 to 84 and the hip fracture incidences for the corresponding age and sex groups for each year between 1998–2002. Data on walking ability status for the population was extracted from consecutive nationwide surveys on health behavior among older people conducted by the National Public Health Institute [52]. Linear regression analysis showed that the prevalence of individuals unable to walk outdoors explained 97.5% of the variation in the hip fracture incidence. In conclusion, the hypothesized association between hip fracture incidence and prevalence of (disability-related) hip fracture risk factors was not falsified, and deserves further examination.

## Conclusion

This study evaluated the feasibility of producing information for the purposes of monitoring hip fracture incidence based on Finnish administrative register data. This type of information production is not a simple task, but requires creative use of rather complicated methodology. It was shown that straightforward use of register data results in biased estimates of the numbers of hip fractures, and that even the sophisticated use of data is dependent on the more or less ambiguous definitions of actual hip fractures.

In this study, the definition of hip fracture was linked to population aging. This reasoning resulted in the suggestion that hip fracture-related disability has certain interesting methodological similarities to the event of death. Moreover, an example from the results empirically supported this interpretation by demonstrating that hip fracture incidence is associated with a certain type of aging-related disability. Actually it seems that hip fracture incidence is directly proportional to the prevalence of population level disability. In this sense, hip fracture incidence trends also reveal the more general disability trends of the population.

Methodologically, while using register-based data, the determination of first aging-related hip fractures is easier than the determination of all hip fractures. Moreover, the detection of the first aging-related hip fracture event closely resembles the determination of the starting point of the hip fracture care episode. A method was developed based on an observed (and expected) hazard function, that allows probabilistic justification for the starting point of the care episode. This method is applicable in the general care episode approach for a wide variety of health problems regardless of the intention to use care episodes in incidence calculations.

Furthermore, three different techniques for risk factor (and corresponding risk population) extraction were demonstrated. Finnish administrative data makes it possible to derive data for a rather detailed population level risk factor stratification, but such a feature has certain more or less data-specific limitations which can be partly avoided by developing methodological solutions to the encountered problems. One area requiring further work that is beyond the scope of this study is the more careful estimation of hip fracture prevalence in Finland.

Finally, this study tries to demonstrate that the traditional methodological paradigm with an assumption of theory-driven data collection and fixed methods has limited suitability for data analyses that utilize secondary data. Two main ideas should be noted. First is the empirical justification of theory-driven operationalized definitions, and second is the theoretical model that is compatible with theory-driven as well as data-driven approaches. For example, in this study register data gave only uncertain estimates for the number of all hip fractures, so the definition and theory underlying the hip fractures had to be revised. After theory revision, empirical justification for the extraction of risk factors was utilized, which finally gave results that supported the revised theory. In fact, it may be difficult to make a clear distinction between definitions, preliminary analyses justifying these definitions, and actual results of the study. In conclusion, it seems that the more detailed (secondary) the available data is, the more alternative perspectives are needed in the analyses of such data and in the reporting of the results of the analyses.

## Competing interests

The author(s) declare that they have no competing interests.

## Authors' contributions

RS carried out the whole research process.
